# Nephro-toxic effects of intraperitoneally injected EGCG in diabetic mice: involvement of oxidative stress, inflammation and apoptosis

**DOI:** 10.1038/srep40617

**Published:** 2017-01-18

**Authors:** Nora O. Abdel Rasheed, Lamiaa A. Ahmed, Dalaal M. Abdallah, Bahia M. El-Sayeh

**Affiliations:** 1 Department of Pharmacology and Toxicology, Faculty of Pharmacy, Cairo University, Egypt

## Abstract

Epigallocatechin gallate (EGCG) has been studied for its beneficial effects. However, some case reports have associated EGCG supplementation with hepato-toxicity. In the present study, we investigated the possible nephro-toxic effects of EGCG in diabetic mice. Streptozotocin (150 mg/kg, i.p.) was injected in mice for diabetes induction. EGCG (100 mg/kg/day, i.p.) was then given for 4 days. The administration of EGCG to diabetic mice caused 60% mortality with no death recorded in other groups. Blood samples were collected for estimation of serum cystatin C, neutrophil gelatinase-associated lipocalin and blood urea nitrogen. Animals were then sacrificed and kidneys were rapidly excised for estimation of oxidative stress markers (NADPH oxidase, reduced glutathione, total antioxidant capacity, nuclear factor erythroid 2-related factor 2, heat shock protein 90, hemeoxygenase-1), as well as inflammatory markers (nuclear factor kappa-B and tumor necrosis factor-α). Administration of EGCG to diabetic mice showed significant elevation in serum cystatin C and neutrophil gelatinase-associated lipocalin, marked increase in oxidative stress and inflammatory states in addition to marked over expression of active caspase-3. Histopathological examination confirmed EGCG induced renal damage in diabetic mice. In conclusion, despite of its well known favorable effects, EGCG could paradoxically exhibit nephro-toxic effect in the presence of diabetes.

Epigallocatechin-3-gallate (EGCG) is the most abundant and well studied catechin in green tea^1^. It has been extensively studied for its beneficial effects as potent anti-oxidant and anti-inflammatory agent in the treatment and prevention of several chronic diseases including cancer, heart diseases, obesity, diabetes, and neurodegenerative diseases^2^,^3^,^4^. However, the use of EGCG based supplements as weight reducing agents has been implicated in liver failure^5^. Moreover, case reports with recurrent episodes of acute hepatitis have been associated with consumption of green tea extract^6^. Most of these reports indicate a correlation between hepato-toxicity and the presence of predisposing factors, such as administration of pharmaceutical drugs or supplements or the presence of other diseased conditions as lipopolysaccharide (LPS) induced fever^7^. It was found that a single high intragastric dose of EGCG did not show hepato-toxicity while combination of single dose EGCG and single dose LPS lead to liver toxicity^7^. Moreover, high doses of EGCG were found to induce toxicity in liver, kidneys and intestine in experimental animals^8^,^9^. EGCG has shown different patterns of toxicity in various animal species. It was found that Teavigo, a highly purified green tea extract, (500 mg/kg, p.o) caused proximal tubule necrosis and elevated serum bilirubin in dogs, whereas oral administration of Teavigo (2000 mg/kg, i.g.) to rats resulted in 80% mortality. Hepatotoxcity in mice was also reported following i.p administration of EGCG^8^. Mice represent an excellent animal model for the study of possible toxicological effects of EGCG due to similar EGCG bioavailability and biotransformation in both mice and human than rat^10^,^11^,^12^.

Diabetes mellitus is a chronic metabolic disorder which has been increasing steadily all over the world. It is characterized by persistent hyperglycemia that can be hazardous to many body organs. Kidneys are one of the important organs affected by diabetes. Moreover, diabetic nephropathy is the most common cause of chronic kidney disease^13^. Hyperglycemia is also known to increase oxidative stress in addition to decrease in antioxidant mechanisms in diabetic patients which may further augment oxidative stress^14^,^15^. This oxidative stress status could trigger an inflammatory cascade that leads eventually to cell death and apoptosis. It is known that the effect of natural products may be different if the health of the subject is compromised or affected by predisposing factor^16^. Thus, diabetes could be used as a representative model for the study of EGCG induced toxicity. The aim of the present study was to test the hypothesis of EGCG possible nephro-toxic effect in diabetic mice and the mechanism involved in this process.

## Materials and Methods

### Animals

Male Swiss albino mice weighing 25–30 g were obtained from the animal facility of Faculty of Pharmacy, Cairo University. Mice were housed under controlled temperature (25 ± 8 C) and constant light cycle (12 h light/dark) and allowed free access to a standard rodent chow diet and water. The investigation complies with the Guide for Care and Use of Laboratory Animals published by the US National Institutes of Health (NIH Publication No. 85–23, revised 2011) and was approved by the Ethics Committee for Animal Experimentation at Faculty of Pharmacy, Cairo University (PT 1271).

### Chemicals

Teavigo, a highly purified extract containing 94% EGCG, was obtained from DSM Nutritional Products, Switzerland and streptozotocin was purchased from Sigma–Aldrich, USA.

### Experimental Design

Mice were randomly divided into 4 groups 8 mice each except group 4 in which 15 mice were used: Group 1 received saline served as normal control. Group 2 received a single dose of STZ (150 mg/kg, i.p) used as diabetic control^17^. Group 3 received EGCG (100 mg/kg, i.p) dissolved in saline for 4 days. Group 4 received STZ as in group 2 then the diabetic animals were injected after 48 hrs with EGCG as in group 3. Diabetes was confirmed by using One Touch Select glucometer (Life Scan, USA). After 48 hrs of STZ injection, animals with blood glucose level ≥200 mg/dl were selected as diabetic animals.

Different doses of EGCG (50, 100, 150 and 200 mg/kg, i.p) were investigated to detect the highest safe dose in normal animals. EGCG 50 and 100 mg/kg did not affect the survival while EGCG 150 and 200 mg/kg caused 40% and 100% mortality, respectively. All doses were given i.p for 4 days and the survival percent, as well as serum cystatin C were recorded compared to normal control. EGCG (50 mg/kg) showed lower level of serum cystatin C versus normal control which indicates its protective role. EGCG (150 mg/kg) showed significantly higher cystatin C levels compared to normal control which indicates its nephro-toxic potential, whereas EGCG (200 mg/kg) killed all injected animals which confirms that it’s a lethal dose. EGCG (100 mg/kg) was the only dose that showed no alteration in serum cystatin C levels compared to normal mice. Accordingly, EGCG (100 mg/kg) was used in the main study (see Supplementary information).

### Preparation of samples

At the end of the experiment, blood was collected under anesthesia from the retro-orbital sinus using non heparinized capillary tubes for estimation of serum cystatin C, serum neutrophil gelatinase-associated lipocalin (NGAL) and blood urea nitrogen (BUN). Animals were then euthanized and kidneys were rapidly excised, washed with ice-cold saline, dried and weighed. Kidneys were homogenized in saline using a homogenizer (Heidolph Diax 900, Germany) to prepare 10% homogenate. Protein content was estimated in the resultant homogenate using the method of Lowry *et al*.^18^. The resultant homogenates were used for determination of oxidative stress and inflammatory markers.

For each group, two kidneys were used from each animal; one for biochemical examination and the other for histological examination.

### Biochemical examination

Enzyme-linked immunosorbent assay (ELISA) kits were used to estimate serum cystatin C (R&D Systems, USA), neutrophil gelatinase-associated lipocalin (NGAL), NADPH oxidase, nuclear factor erythroid 2-related factor 2 (Nrf2) (Cusabio, China), hemeoxygenase-1(HO-1) (Enzo Life Sciences, Egypt), heat shock protein 90 (HSP 90) (Bioassay™, USA), nuclear factor kappa-B (NF-κB) (Eiaab, China) and tumor necrosis factor-α (TNF-α) (Raybio, USA). Reduced glutathione (GSH) and blood urea nitrogen (BUN) were estimated using glutathione reduced and blood urea nitrogen colorimetric kits, respectively (Biodiagnostic, Egypt). Total antioxidant capacity (TAC) was assessed by antioxidant assay kit (Zenbio, USA).

### Histological examination

At the end of the experiment, kidneys were isolated, rinsed in ice-cold saline and immediately fixed in 10% formalin. The specimens were processed for paraffin embedding and 5 μm sections were prepared.

### Immunohistochemical examination of active caspase-3

Active caspase 3 examination was carried out using paraffin embedded tissue sections of 5 μm thickness. To reveal the antigens, sections were pre-treated with the proteolytic enzyme proteinase K (Dako, Copenhagen, Denmark) and were then washed in phosphate buffered saline (PBS) for 5 min. Subsequently, sections were incubated with a primary antibody against caspase-3 (Novus biologicals, USA) for 60 min at 37 °C. After washing in PBS, a secondary antibody (Novus biologicals, USA) was applied for 60 min. The reaction was visualized with 3,3′-diaminobenzidine (DAB) chromagen (Dako, Copenhagen, Denmark). The slides were then counterstained with hematoxylin, mounted and examined. For quantification of apoptosis percentage, the percentage of brown staining, indicating active caspase 3 expression, was estimated using light microscope with an image analyzer (Leica Qwin 550, Germany) (magnification x 400) from 5 random selected fields in each section and averaged.

### Light microscopic examination

For light microscopic examination, the sections were stained with haematoxylin–eosin (H&E) and examined by light microscope (magnification x400).

### Statistical analysis

All data obtained were presented as mean ± SEM. Results were analyzed using one way analysis of variance test (one-way ANOVA) followed by Tukey-Kramer multiple comparisons test. Statistical analysis was performed using GraphPad Prism software (version 6.01). For all the statistical tests, the level of significance was fixed at *P* < 0.05.

## Results

### Mortality

Administration of EGCG to diabetic mice showed 60% mortality, whereas no mortality was recorded in the other groups.

### Body weight

Administration of EGCG to diabetic mice showed no significant reduction in body weight compared to diabetic animals (Table 1).

### Kidney biomarkers

Serum cystatin C and NGAL were assessed as early markers of kidney injury. Administration of EGCG to diabetic mice markedly increased serum levels of both cystatin C and NGAL compared to diabetic mice which confirmed that EGCG potentiated diabetes induced kidney damage. BUN was assessed as a surrogate marker associated with increased severity of renal and/or systemic illness. Diabetic animals receiving EGCG showed no significant alteration in BUN level as compared to diabetic and normal mice which indicates that the animals were not in a lethal condition. EGCG group showed no significant alteration in serum parameters compared to normal group (Tables 2 and 3).

### Oxidative stress markers

**Figure 1:**
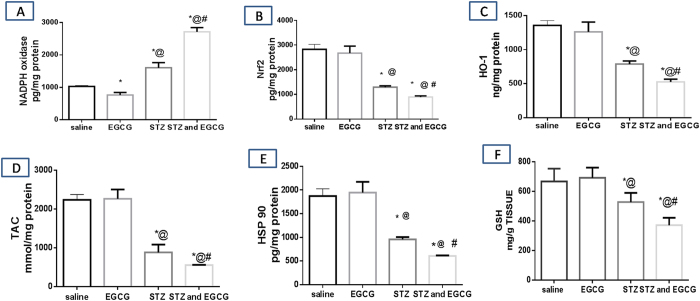
Effect of EGCG on STZ-induced changes in (**A**) NADPH oxidase, (**B**) Nrf2, (**C**) HO-1, (**D**) TAC (**E**) HSP 90 and (**F**) GSH. Each value represents the mean of 6–8 experiments ± S.E.M. ^*^P < 0.05 vs. normal, ^@^*P* < 0.05 vs. EGCG, ^#^*P* < 0.05 vs. STZ.

Oxidative stress status was evaluated by the measurement of NADPH oxidase content as a major source of reactive oxygen species (ROS) as well as TAC and GSH as markers of enzymatic and non-enzymatic antioxidant defense systems. Nrf2 and its dependant enzyme; HO-1 were also assessed together with HSP 90 as they normally help the body to adapt to stress in order to prevent stress induced damage. In normal mice, EGCG administration significantly decreased the NADPH oxidase content with no significant alteration in other oxidative stress markers. On the other hand, diabetic mice receiving EGCG have shown deteriorated oxidative stress status with significant increase in NADPH oxidase and significant decrease in TAC, Nrf2, HO-1 and HSP 90. This indicates that EGCG administration in normal mice suppressed reactive oxygen species (ROS) production by decreasing NADPH oxidase content which is responsible for superoxide generation. On the other hand, EGCG administration to diabetic mice potentiated ROS production and also ameliorated the adaptive mechanisms that normally suppress ROS generation. This was indicated by significant increase in NADPH oxidase content and down regulation of TAC, Nrf2, HO-1 and HSP 90 (Fig. 1).

### Inflammatory markers

**Figure 2:**
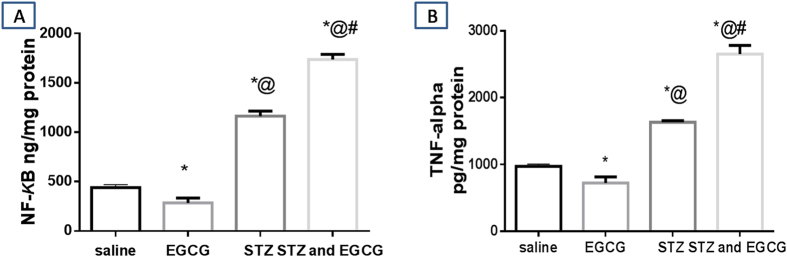
Effect of EGCG on STZ-induced changes in (**A**) NfκB and (**B**) TNF-α. Each value represents the mean of 6–8 experiments ± S.E.M. ^*^P < 0.05 vs. normal, ^@^*P* < 0.05 vs. EGCG, ^#^*P* < 0.05 vs. STZ.

Oxidative stress activates NF-κB which regulates the expression of several genes that are involved in the production of inflammatory cytokines, including TNF-α. In normal mice, EGCG administration caused significant decrease in both NF-κB and TNF-α while both parameters were markedly increased in diabetic animals which received EGCG indicating a severe inflammatory status (Fig. 2).

### Histological examination

#### Immunohistochemical examination of active caspase-3

**Figure 3:**
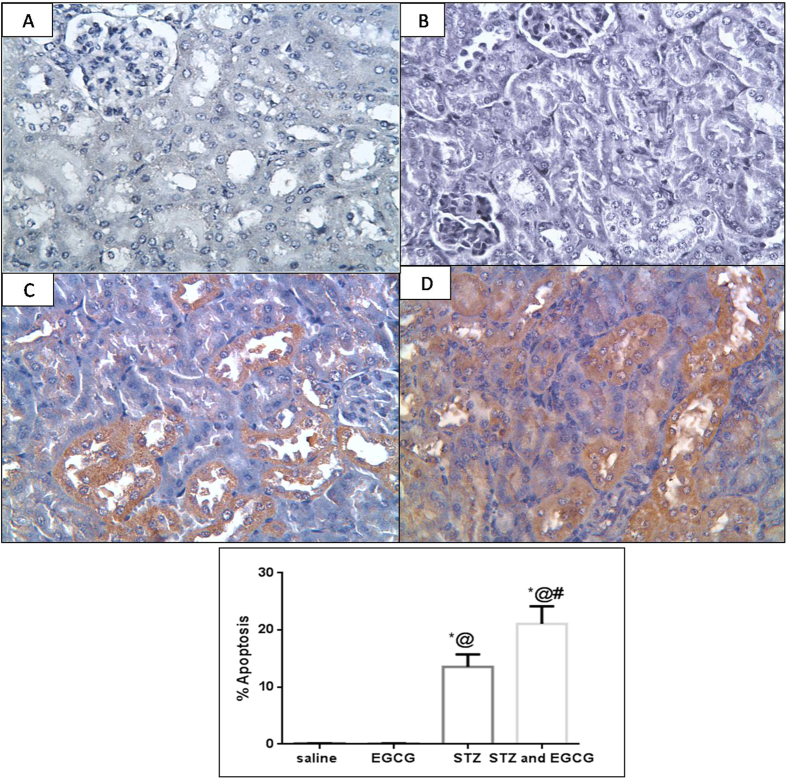
Effect of EGCG on STZ- induced changes in immunohistochemical expression of active caspase-3 expression. (**A**) (saline) showing normal tubules and glomeruli with –ve immunostaining for active caspase (x400), (**B**) (EGCG) showing normal tubules and glomeruli with –ve immunostaining for active caspase (x400), (**C**) (STZ) showing degenerated tubules with focal immunostaining of the epithelium for active caspase (x400) and (**D**) (STZ and EGCG) showing degenerated tubules with vacuolated epithelium showing wide immunostaining for active caspase (x400). (**E**) Each value represents the mean of 3 experiments ± S.E.M. ^*^P < 0.05 vs. normal, ^@^*P* < 0.05 vs. EGCG, ^#^*P* < 0.05 vs. STZ.

Oxidative stress and inflammation lead to active caspase-3 increased expression indicating the incidence of apoptosis. EGCG group showed no alteration from normal control regarding active caspase-3 expression. Diabetic mice receiving EGCG showed significant elevation in active caspase-3 expression which was indicated by marked elevation of percentage apoptosis, whereas diabetic mice showed a significantly lower apoptotic percentage (Fig. 3).

### Light microscopic examination

**Figure 4:**
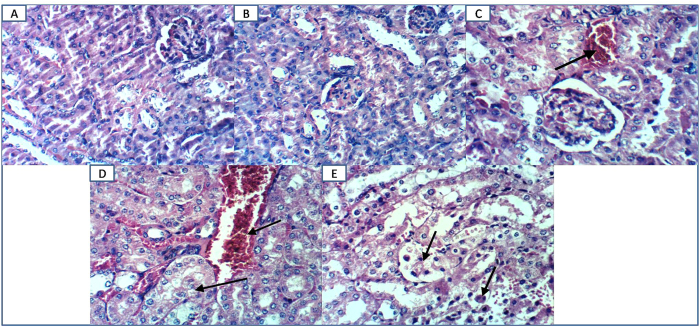
Effect of EGCG on STZ- induced changes in histopathological examination. (**A**) Kidney of mouse from control group showing no histopathological changes (H & E X 400) (**B**) kidney of mouse from EGCG group showing no histopathological changes (H & E X 400). (**C**) Kidney of mouse from diabetic group showing moderate congestion of renal blood vessel (H & E X 400) (**D**). Kidney of mouse from diabetic group receiving EGCG showing severe congestion of renal blood vessel and vacuolation of renal tubular epithelium (H & E X 400). (**E**) Kidney of mouse from diabetic group receiving EGCG showing necrosis of epithelial lining renal tubules (H & E X 400).

Under normal conditions, EGCG administration showed no histopathological alteration compared to normal mice. Diabetic mice showed moderate congestion of renal blood vessels. EGCG administration to diabetic mice caused severe congestion of renal blood vessels and vacuolation of renal tubular epithelium in addition to necrosis of epithelial lining renal tubules. This confirms that EGCG potentiates diabetic induced kidney damage (Fig. 4).

## Discussion

EGCG, the main and most significant polyphenol in green tea, has shown numerous health promoting effects acting through different pathways, as antioxidant, anti-inflammatory and anti-atherogenic agent^19^,^20^,^21^. However, some case reports have linked hepato-toxicity to EGCG supplementation especially in presence of contributing factors that can evoke EGCG toxicity such as fever^7^. In most of the toxicity reports, high doses of dietary supplements containing concentrated or purified tea preparations were used. Moreover, the doses at which toxicity was observed were much higher than those typically delivered by normal tea consumption, but they are more readily achievable in the context of tea-based dietary supplements^8^. In the present study, we investigated the possible nephro-toxic effects of EGCG in diabetic mice. The administration of EGCG or STZ alone did not affect the animal survival while EGCG lead to 60% mortality in diabetic animals yet it caused no significant reduction in the body weight of diabetic animals compared to diabetic mice. Moreover, kidney injury was confirmed by significant increase in both serum cystatin C and NGAL levels. The significant correlation between NGAL, cystatin C and glomerular filtration rate decline supported the prognostic role of both markers in revealing early structural renal damage before an overt renal impairment becomes evident^22^. Diabetic mice receiving EGCG showed no significant alteration in BUN level compared to diabetic and normal mice which indicates that the animals were not in a lethal condition as the elevation in BUN is known to have a negative impact on survival^23^. Concerning oxidative stress parameters, EGCG group showed no significant alteration from normal mice regarding TAC, GSH, Nrf2, HO-1 and HSP 90. Interestingly, NADPH oxidase content was significantly improved compared to normal mice. On the other hand, diabetic animals receiving EGCG showed marked deterioration in oxidative stress state compared to diabetic mice. This was indicated by significant increase of NADPH oxidase content which is the major source of ROS production^24^. TAC and GSH were massively decreased indicating attenuation of defense mechanisms. Since this study revealed excessive reduction of both GSH and TAC by EGCG in diabetic mice kidneys, hence it might be speculated that such effects are a consequence of NADPH oxidase increment, in addition to the reported pro-oxidant effect of EGCG which contributes to the formation of huge amounts of ROS^25^. Furthermore, EGCG is known to cause oxidative damage in the presence of transition metals, such as copper and iron^26^. Meanwhile, diabetes is known to be associated with increased levels of these metals^27^,^28^. Therefore, diabetes induced increase in copper and iron levels might be implicated in EGCG toxicity. Apart from the involvement of NADPH oxidase, Nrf2, HO-1 and HSP 90 down regulation were also implicated in EGCG toxicity. When oxidative stress increases under normal conditions, it induces adaptive defense systems for oxidative stress and proteo stress, which are specifically sensed by Keap 1 and HSP90β, respectively^29^. This activates the key transcription factors, Nrf2 and heat shock factor 1(HSF1) which induce expression of numerous genes for adaptation such as HO-1^29^. However, in the present study, administration of EGCG to diabetic mice increased oxidative stress to an extent that overwhelmed that adaptive mechanism leading to down regulation in Nrf2, HSP90 and HO-1 expression. Our results are in accordance with other studies in which high doses of EGCG reduced the expressions of Nrf2-dependent antioxidant enzymes, including HO-1^29^,^30^. Moreover, the present study is consistent with the previous finding that HSP90 was notably down-regulated in the kidneys of mice that received high doses of green tea polyphenols in diet^9^.

Oxidative stress is also known to induce the expression of redox sensitive transcription factor NF-κB which plays a crucial role in triggering the expression of inflammatory cytokines, such as TNF-α^31^. Meanwhile, this pro-inflammatory cytokine was documented to increase the transcriptional activity of NF-κB which increases oxidative stress in turn^32^. Beside the depletion of antioxidant defenses as previously anticipated herein, massive increase of renal NF-κB, as well as TNF-α contents above diabetic control values were detected in diabetic mice receiving EGCG. However, both parameters were significantly improved in EGCG group compared to normal mice. Therefore, EGCG-induced increment in TNF-α content observed in presence of diabetes can augment oxidative stress and inflammation. Other explanations could involve the decrease in HSPs that exerts anti-inflammatory activity by blocking NF-κB activation^33^. Hence, excessively reduced HSP90 and HO-1(HSP 32) in EGCG/STZ group could have augmented NF-κB activity and inflammation to postulate that EGCG potentiated diabetes-induced inflammation is not only linked to oxidative stress but also through HSP90 and HO-1 alleviation. In the present study, significant increase of renal percentage of apoptosis was observed in diabetic mice receiving EGCG evidenced by widely distributed immunohistochemical staining of active caspase-3. Both of TNF-α and ROS activate the extrinsic and intrinsic programmed death pathways, respectively^34^. The increased pro-inflammatory cytokine together with reduced levels of GSH, TAC, Nrf2, HO-1 and HSP90 that signify overwhelmed antioxidant defenses with the activation of NOX could clarify the overexpression of caspase-3 in this study after EGCG administration to diabetic mice. Finally, histopathological examination revealed necrosis of epithelial lining renal tubules, severe congestion of renal blood vessels and vacuolation of renal tubular epithelium in diabetic animals receiving EGCG. On the other hand, diabetic animals showed only moderate congestion of renal blood vessels. These microscopical data emphasize that EGCG has a deleterious impact on the kidney of diabetic animals that overweigh those of hyperglycemia alone.

Most of the previous studies suggest that high doses of EGCG can induce toxicity in the liver, kidneys, and intestine of normal animals^8^,^9^. However, the current investigation showed that EGCG toxicity can occur even on using non toxic doses. In other words, EGCG toxicity might ensue in the presence of certain diseases, namely diabetes, even though no toxicity was recorded in normal animals receiving the same dose.

## Conclusion

Despite the reported beneficial effects of EGCG, sometimes under certain pathological conditions, such as diabetes, it could behave paradoxically. The current findings elaborate that EGCG produces nephro-toxicity in diabetic mice via exaggerated oxidative stress, inflammation, and apoptosis triggered by hyperglycemia. Therefore, it is recommended that diabetic patients should consume EGCG based supplements with caution until further studies assert its toxicity in human.

## Additional Information

**How to cite this article:** Abdel Rasheed, N. O. *et al*. Nephro-toxic effects of intraperitoneally injected EGCG in diabetic mice: involvement of oxidative stress, inflammation and apoptosis. *Sci. Rep.* **7**, 40617; doi: 10.1038/srep40617 (2017).

**Publisher's note:** Springer Nature remains neutral with regard to jurisdictional claims in published maps and institutional affiliations.

## Supplementary Material

Supplementary Information

## Figures and Tables

**Table 1:** Effect of EGCG administration on STZ induced changes in body weight.

Groups	Body weight (gm)
Saline (normal)	27.00 ± 0.7071
EGCG	27.00 ± 0.9487
STZ	23.20 ± 0.5831^*@^
STZ and EGCG	22.80 ± 0.7348^*@^

Each value represents the mean of 6–8 experiments ± S.E.M. ^*^P < 0.05 vs. normal, ^@^*P* < 0.05 vs. EGCG.

**Table 2:** Effect of EGCG on STZ-induced changes in serum cystatin C and serum NGAL.

Groups	NGAL (ng/mL)	Cystatin C (pg/mL)
Saline (normal)	25.29 ± 1.073	28.08 ± 1.147
EGCG	27.55 ± 0.7909	29.95 ± 1.046
STZ	85.84 ± 2.009^*@^	95.45 ± 1.737^*@^
STZ and EGCG	138.7 ± 4.332^*@#^	148.5 ± 3.521^*@#^

Each value represents the mean of 6–8 experiments ± S.E.M. ^*^P < 0.05 vs. normal, ^@^*P* < 0.05 vs. EGCG, ^#^*P* < 0.05 vs. STZ.

**Table 3:** Effect of EGCG administration on blood urea nitrogen (BUN) in normal and diabetic mice.

Groups	BUN (mg/dL)
Saline (normal)	38.20 ± 0.6633
EGCG	37.20 ± 1.020
STZ	39.40 ± 0.8124
STZ and EGCG	38.80 ± 0.9695

Each value represents the mean of 6–8 experiments ± S.E.M.
