# Supplementary material for: Nephro-toxic effects of intraperitoneally injected EGCG in diabetic mice: involvement of oxidative stress, inflammation and apoptosis
Source: Sci Rep. 2017 Jan 18;7:40617. doi: 10.1038/srep40617 (PMC5241811; doi:10.1038/srep40617)
Supplement: Supplementary Information [file srep40617-s1.pdf]

Nephro-toxic effects of intraperitoneally injected EGCG in diabetic mice: involvement of oxidative stress, inflammation and apoptosis

Authors:

***Nora Osama Abdel Rasheed[1], Lamiaa Ahmed Ahmed[2], Dalaal M. Abdallah[3], Bahia M. El-Sayeh[4]**

Corresponding author: Nora Osama Abdel Rasheed[1]

1.Department of Pharmacology and Toxicology , Faculty of Pharmacy, Cairo University, Egypt.

2. Department of Pharmacology and Toxicology , Faculty of Pharmacy, Cairo University, Egypt.

3. Department of Pharmacology and Toxicology , Faculty of Pharmacy, Cairo University, Egypt.

4. Department of Pharmacology and Toxicology , Faculty of Pharmacy, Cairo University, Egypt.

Supplementary table 1: Effect of different doses of EGCG (50,100,150 and 200 mg/kg) on percentage of mortality, serum cystatin C.

	**Normal conrol (saline)**	**EGCG 50**	**EGCG100**	**EGCG150**	**EGCG200**
%mortality	0%	0%	0%	40%	100%
Serum cystatin C pg/mL	33.80±1.463	23.63±0.8715*	32.30±0.8602	68.33±1.829*	Not estimated as all animals died

Each value represents the mean of 5-8 experiments ± S.E.M.
* Significantly different from normal group at $P < 0.05$
